# Associations of Temporal Eating Patterns with Nutrient Intake Variability and Diet Quality Among Japanese Female Mobile Application Users

**DOI:** 10.3390/nu18060957

**Published:** 2026-03-18

**Authors:** Ariko Umezawa, Noriko Sato, Hiiro Terasaki, Yu Tahara, Shigenobu Shibata

**Affiliations:** 1 Department of Nutritional Science, Faculty of Food and Nutritional Sciences, Japan Women’s University, Tokyo 112-8681, Japan; umezawaa@fc.jwu.ac.jp; 2 Division of Food and Nutrition, Graduate School of Human Sciences and Design, Japan Women’s University, 2-8-1 Mejiro-dai, Bunkyo-ku, Tokyo 112-8681, Japan; terasaki.hiiro@gmail.com; 3 Graduate School of Biomedical and Health Sciences, Hiroshima University, Kasumi, Minami-ku, Hiroshima City 734-0037, Japan; yutahara@hiroshima-u.ac.jp (Y.T.); shibatas@hiroshima-u.ac.jp (S.S.); 4 Faculty of Home Economics, Aikoku Gakuen Junior College, NishiKoiwa, Edogawa-ku, Tokyo 133-0057, Japan

**Keywords:** mealtime irregularity, chronotype, dietary habits, diet quality, nutrient intake variability

## Abstract

**Background/Objectives:** Although lifestyle patterns, including sleep and meal timing, have been associated with diet quality, previous studies have frequently relied on time-averaged data, which can obscure day-to-day intake variability. Using data from a food-logging mobile application, we aimed to elucidate the association between mealtime irregularity and nutrient intake variability. Furthermore, we explored whether the association between meal timing and diet quality differs depending on mealtime regularity. **Methods:** Chrononutritional characteristics were calculated for 742 female mobile application users who provided daily dietary records for approximately 1 month. Nutrient intake variability was evaluated using the coefficient of variation (CV). Diet quality was assessed based on the energy-adjusted ratio of nutrient intake to the reference values from the Dietary Reference Intakes for Japanese. Generalized additive models adjusted for age, body mass index, and physical activity were employed for analyzing associations. **Results:** Greater mealtime irregularity was associated with higher CVs in the daily intake of most nutrients investigated. Regarding diet quality, a marginal interaction was observed between mealtime regularity and dinner timing (*p* for interaction = 0.062). Specifically, the irregular mealtime group demonstrated a significant linear decline in diet quality with later dinner times (*p* for trend = 0.0112), whereas no significant decline was observed in the regular mealtime group (*p* for trend = 0.5219). **Conclusions:** Our findings suggest that mealtime regularity, alongside meal timing, is a significant factor involved in nutrient intake variability and diet quality, underscoring its significance as a health-related behavior in chrononutrition.

## 1. Introduction

Chrononutritional habits, including an early chronotype, avoiding excessively late meals, and regular meal timing, have been suggested to be associated with healthier food and nutrient consumption [1,2,3,4]. These habits may promote the synchronization of central and peripheral clocks, thereby physiologically contributing to health maintenance [5,6]. However, most previous studies have focused on average lifestyle patterns, and the association between day-to-day regularity of mealtime and diet quality and regularity (low day-to-day variability in nutrient intake) remains largely unclear.

This study focuses on two aspects of evaluating a healthy diet. The first is “diet quality”, which refers to the overall nutrient or food intake without deficiency or excess [7,8]. Diet quality can be assessed by the adequacy of intake in multiple recommended nutrients and the restriction of excessive nutrient intake based on the reference daily value specified in the Dietary Reference Intakes for the Japanese population (DRIs-J). “Dietary intake consistency”, which reflects low day-to-day nutrient intake variability, represents the second aspect. Achieving stable and sufficient intake of recommended nutrients (e.g., dietary fiber, minerals, and vitamins) and minimizing the day-to-day variation in nutrients (e.g., salt and saturated fatty acids [SFAs]) are crucial for maintaining homeostasis and promoting gut health [9,10,11]. Elucidating the association among nutrient intake variability (coefficient of variation [CV] of daily intake), diet quality, and chrononutrition-related characteristics could have significant implications for lifestyle-related disease prevention.

Our previous study using food-logging mobile application data [12] revealed an association between mealtime irregularity and greater day-to-day variability (CV) in energy intake (EI), independent of total daily intake. Additionally, we noted a positive association between breakfast time irregularity and body mass index (BMI) among older females. These findings suggest that irregular mealtimes can be associated with poorer diet status. Therefore, this study aims to clarify how chrononutritional characteristics, including conventional factors (e.g., chronotype and habitual meal timing) and day-to-day mealtime regularity, are associated with diet quality and regularity.

## 2. Materials and Methods

### 2.1. Study Population and Design

This study was a retrospective cross-sectional analysis conducted using dietary intake data extracted from asken, a popular food-logging mobile health application in Japan, as previously described [12]. The asken application is broadly employed across different age groups, and several studies have confirmed the reliability of its self-reported food log data for dietary habit assessment [13,14,15,16]. This study utilized baseline data from the “Exercise Intervention Study Using the Mobile Health Application Asken.” This study was approved by the Ethics Review Committee on Research with Human Subjects at Waseda University (approval number: 2020-046) and conducted following the Declaration of Helsinki. All participants provided informed consent.

Overall, 5619 participants had food log data at baseline (September–October 2021). Of these participants, 1551 who logged meals for at least 25 days per month were selected. We excluded implausible data (e.g., unrealistically high nutrient intake values, impossible meal timing sequences, or meal intervals shorter than 15 min; *n* = 11), shift workers (*n* = 253), and participants who used only default mealtimes (*n* = 185). Of the 1102 participants who met the abovementioned eligibility criteria, 742 females were included in this study.

### 2.2. Data Collection and Processing

The method for collecting dietary record data was detailed in our previous study [12]. In brief, a meal was defined as an intake of at least 50 kcal (210 kJ). The asken application categorizes meals as either “breakfast”, “lunch”, “dinner”, or “snack”, and users select one of these categories when recording their intake. As the asken application enables mealtime entry only for the three main meals (breakfast, lunch, and dinner) and not for “snacks”, this study analyzed mealtimes and their irregularity for the three main meals.

Data on age, sex, height, weight, shift work status, and lifestyle factors were collected through an online self-administered questionnaire at baseline. Moreover, average monthly EI and body weight data were collected 1 month later (November 2021). Physical activity was evaluated using the short version of the Physical Activity Questionnaire [17] and expressed in metabolic equivalent of task (MET)-hours per week. Circadian sleep habits were assessed using the short version of the Munich Chronotype Questionnaire [18], and the sleep-corrected midpoint on free days (MSFsc) was calculated as previously described [12]. Participants were classified into morning (MSFsc [h] < 2.57), intermediate (2.57 ≤ MSFsc [h] < 3.75), and evening (3.75 ≤ MSFsc [h]) chronotypes.

Regarding mealtimes, the 1-month average times for breakfast and dinner were calculated. Participants were categorized into four groups on the basis of their breakfast time: (1) before 7:00 a.m., (2) from 7:00 a.m. to before 8:00 a.m., (3) from 8:00 a.m. to before 9:00 a.m., and (4) 9:00 a.m. or later. Similarly, they were classified into five groups on the basis of their dinner time: (1) before 6:00 p.m., (2) from 6:00 p.m. to before 7:00 p.m., (3) from 7:00 p.m. to before 8:00 p.m., (4) from 8:00 p.m. to before 9:00 p.m., and (5) 9:00 p.m. or later.

Day-to-day mealtime irregularity was assessed using the composite phase deviation (CPD) metric as previously described [12,19]. CPD is calculated based on both the difference in mealtime from the previous day (day-to-day stability) and the deviation from the individual’s mean mealtime (Figure S1). As the CPD of the three main meals exhibited a high correlation [12], participants with a CPD of ≥1.0 h for any meal were categorized into the irregular mealtime group. We selected CPD ≥ 1.0 h as the threshold for mealtime irregularity because, from a practical standpoint, a day-to-day mealtime variation of around 30 min is typically considered a normal fluctuation. Conversely, a deviation of more than 1.0 h may represent a more substantial variability that is readily recognized as irregular in daily life. To validate this cutoff, a sensitivity analysis was conducted by dividing the participants into three groups: a regular mealtime group (CPD < 0.5 h for all meals), a slightly irregular mealtime group (CPD ≥ 0.5 h for any meal but <1.0 h for all meals), and an irregular mealtime group (CPD ≥ 1.0 h for any meal).

The nutritional value of each meal, including EI, was calculated using the latest 2020 Standard Tables of Food Composition (STFC) in Japan (eighth edition) [20]. Total daily energy or nutrient intake was calculated as the sum of intake from breakfast, lunch, dinner, and snacks. Each individual’s mean snack energy contribution (%E) was calculated as the 1-month average of daily proportion of energy from snacks relative to the total daily energy intake. The coefficient of variation (CV) of snack energy intake was calculated by dividing the standard deviation of daily snack energy intake by its mean. We analyzed nutrient intake only, as we did not assess food group intake based on the STFC food group classification because the necessary information was not available from asken.

Nutrient intake variability was assessed by calculating the CV for the daily intake of energy and 22 nutrients over a 1-month period (Table 1). The CV was determined by dividing its standard deviation by its mean intake. Daily variability in energy and macronutrient intake can disrupt blood glucose homeostasis and metabolic regulation [11]. Furthermore, variability in anti-inflammatory nutrient and antioxidant intake can adversely impact health. The CV for the intake of specific nutrients has recently been reported to be associated with gut microbiota diversity [9] (Table 1). Accordingly, this analysis included these relevant nutrients.

The ratios of nutrient intakes relative to their reference values were computed. This analysis encompassed 17 nutrients with age-specific reference values established in the DRIs-J 2025 [21] (Table 2). Individual nutrient intakes for total daily intake were calculated as 1-month averages. Days with no meal records were treated as missing and excluded from the averaging. All nutrients were derived from the same set of recorded days within each participant. Given that only participants with at least 25 recording days were included, the mean (SD) number of recorded days was high at 30.0 (1.58), ensuring the stability of the averaged data. Subsequently, these intakes were energy-adjusted using the density method to evaluate nutrient intake density independent of the actual EI. The ratios were calculated relative to the reference values corresponding to the age-specific estimated energy requirement following standard methodology by assuming a moderate physical activity level (PAL, Level II) for all participants to standardize for diet quality assessment [21].

Diet quality using a composite score based on the algorithm of the Nutrient-Rich Food Index [22]. The score was calculated using the energy-adjusted intake ratio calculated above. First, the ratios for 15 adequacy components (recommended nutrients) were summed, with each ratio capped at 1.0. This cap ensures that an excessively high intake of one nutrient cannot compensate for the insufficiency of another. Second, for the moderation components (nutrients to be limited), a penalty was calculated as the proportional excess over their reference limits. To determine the final nutritional score, this penalty was subsequently subtracted from the adequacy sum.

### 2.3. Evaluation of Reported EI

The reliability of EI data from dietary assessments is a crucial factor in chrononutrition research. Our study employed food logging via a mobile application, a method less reliant on memory than conventional approaches. Furthermore, the diligence with which participants recorded their daily meals suggests a low misreporting rate.

In this study, we evaluated the validity of reported EI using the Goldberg cut-off [23], which is predicated on the assumption of energy balance where body weight does not change. Because the participant’s body weight at 1 month later was available, we were able to assess the relationship between the reported EI and body weight change. If body weight remains stable, EI is approximately equivalent to energy expenditure (EE). Therefore, a reported EI is deemed acceptable when the EI-to-basal metabolic rate (BMR) ratio falls within the 95% confidence interval (CI) for the participant′s PAL. We calculated BMR using the Ganpule equation [24]. For the estimation of EE, we assumed that the general adult female population engages in sedentary-to-light physical activities; therefore, we applied a representative PAL of 1.55 based on the FAO/WHO/UNU recommendation [25]. The 95% confidence limits for agreement (upper and lower cut-off values) between EI:BMR and PAL were calculated using the values of 15.6%, 8.5%, 15%, and 30 for the within-subject CV in the reported EI, CV of repeated BMR measurements, between-subject variation in PAL, and number of dietary recording days, respectively [23]. Participants were categorized into three groups: low-ratio group (EI:BMR ratio < the lower limit of the 95% CI of the PAL), high-ratio group (EI:BMR ratio > the upper limit of the 95% CI of the PAL), and acceptable-ratio group (all others). Notably, participants in the low-ratio group exhibited weight loss, whereas those in the high-ratio group demonstrated weight gain (Figure S2). This physiological consistency between reporting status and actual weight change supports the plausibility of the reported EIs in this study.

Although assessing the reported EI solely at baseline should be sufficient, discussing its relationship with weight changes after 1 month requires the assumption that the EI remains stable. Therefore, we performed an equivalence test using the two one-sided tests (TOST) procedure to confirm that the mean EI did not substantially vary between baseline and at 1 month later. We defined the equivalence margin as ±155 kcal (approximately 10% of the mean EI). The TOST procedure indicated that the difference was significantly within the equivalence bounds (*p* < 0.0001). Thus, we confirmed the stability of the reported EI and a consistent relationship between the magnitude of the EI:BMR ratio and body weight changes.

### 2.4. Statistical Analysis

Statistical analyses were performed using R (version 4.5.1; R Foundation for Statistical Computing, Vienna, Austria) and Stata (version 18; StataCorp., College Station, TX, USA). Visualizations were constructed using the *ggplot2* package (ver. 4.0.0). The CV of the intake for each nutrient, the ratio of intake to the reference value, and the nutritional score were the variables employed for evaluating diet regularity and quality. Chronotype, breakfast and dinner timing, and mealtime regularity were the chrononutritional variables. Chronotype was categorically coded as follows: 0 = morning type, 1 = intermediate type, and 2 = evening type. The breakfast time group was coded as follows: 0 = before 7:00 a.m., 1 = from 7:00 a.m. to before 8:00 a.m., 2 = from 8:00 a.m. to before 9:00 a.m., and 3 = 9:00 a.m. or later. The dinner time group was coded as follows: 0 = before 6:00 p.m., 1 = from 6:00 p.m. to before 7:00 p.m., 2 = from 7:00 p.m. to before 8:00 p.m., 3 = from 8:00 p.m. to before 9:00 p.m., and 4 = 9:00 p.m. or later. Mealtime irregularity was coded as 0 (regular) and 1 (irregular). Association analyses of chrononutritional variables with dietary variables were performed using generalized additive models (GAMs) implemented with the *mgcv* package (ver. 1.9.3) in R. To handle potential nonlinearity, continuous covariates, including age, BMI, and physical activity (MET-hours/week), were modeled using penalized spline smooth terms, with basis dimensions evaluated and adjusted as necessary. Smoothing parameters were estimated by restricted maximum likelihood (REML). Model diagnostics included inspection of residual plots and Q–Q plots and evaluation of smooth-term adequacy using *gam.check* (including the k-index). Regarding the exposures, dinner timing and mealtime regularity were modeled as ordinal linear terms (ordered categorical factors). To examine whether mealtime regularity may modify the association between nutritional score and dinner timing, we included a standard parametric interaction term between the mealtime regularity and dinner timing in the models. The statistical significance of the potential effect modification was assessed using the *p*-value for this interaction term. We further conducted tests for linear trend across the ordered dinner timing categories based on estimated marginal means calculated with the *emmeans* package (ver.2.0.0). Specifically, we obtained adjusted marginal means from the interaction model and applied linear (polynomial) contrasts to compare the trend slopes in the nutritional score (diet quality) associated with later meal timing between the regular and irregular mealtime groups. A *p*-value of <0.05 was considered statistically significant.

We performed sensitivity analyses by additionally adjusting for snacking behavior proxies—mean snack energy contribution (%E) and day-to-day variability of snack energy intake (CV)—to assess whether unmeasured snack timing might confound the associations between mealtime irregularity (based on the three main meals) and outcomes. These proxies cannot capture snack timing irregularity directly; however, they account for the magnitude and variability of snacking that could be correlated with overall eating pattern irregularity.

Because this study was designed as an exploratory and hypothesis-generating investigation, we did not apply formal adjustments for multiple comparisons (e.g., false discovery rate) to the *p*-values. Consequently, the findings regarding individual nutrients should be interpreted with caution.

## 3. Results

### 3.1. Participant Characteristics

The basic characteristics of the participants are presented in Table 3. As demonstrated in our previous study [12], mealtime regularity was evaluated using the CPD metric for each meal type (breakfast, lunch, and dinner). This evaluation was determined by day-to-day variability in mealtimes, independent of the day of the week or weekday/holiday status. The CPD values for each meal showed high correlation; when variability in one meal was large, variability in the other meals was correspondingly large. Therefore, in this study, participants were categorized into the irregular mealtime group when the CPD value for any meal exceeded 1 and into the regular mealtime group otherwise. The irregular mealtime group exhibited a significantly lower mean age, later wake-up, bedtime, mealtimes, and higher proportion of evening type than the regular mealtime group (Table 3).

To verify the robustness of the cutoff value for defining the irregular mealtime group, a sensitivity analysis was performed using a more graded classification with alternative thresholds (0.5 h and 1.0 h) (Table S1). As a result, sleep/wake times and mealtimes were similar between the regular mealtime group (all meals with CPD < 0.5 h) and the slightly irregular mealtime group (all meals with CPD < 1.0 h but some exceeding 0.5 h). However, the irregular mealtime group (all meals with CPD ≥ 1.0 h) showed significantly delayed times (Table S1).

### 3.2. Distribution Classified by Chronotype, Meal Timing, and Mealtime Regularity

**Figure 1:**
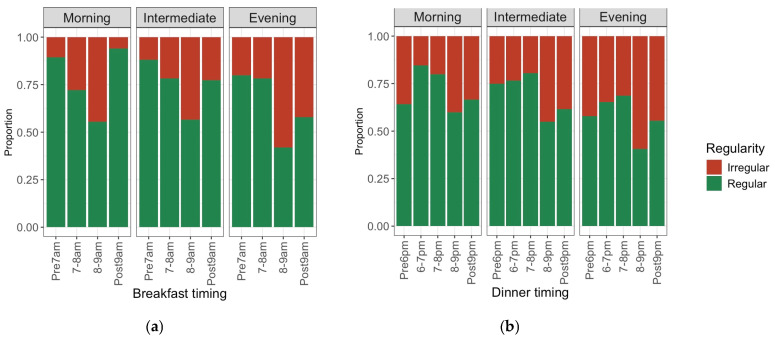
Proportions of mealtime regularity by meal timing faceted by chronotype. (**a**) Proportions of mealtime regularity by breakfast timing and chronotype. (**b**) Proportions of mealtime regularity by dinner timing and chronotype.

We investigated the proportions of the irregular mealtime group across different meal timings. For breakfast timing, the proportion of the irregular group was the highest between 8:00 a.m. and 9:00 a.m. (Figure 1a). For dinner timing, the proportion was high both before 6:00 p.m. and after 8:00 p.m. (Figure 1b). Furthermore, as mentioned above (Table 3), the evening chronotype was more prevalent in the irregular mealtime group. These findings indicate that although chronotype, meal timing, and mealtime regularity are interrelated, the association between timing and regularity is not merely linear. Therefore, we investigated the independent associations of chronotype, meal timing, and regularity as chrononutritional factors with dietary quantity, quality, and intake variability.

### 3.3. EI and Its CV by Chrononutritional Classification

**Figure 2:**
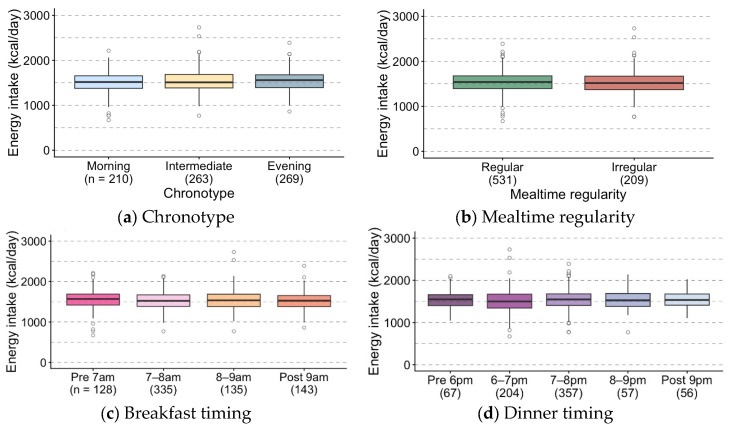
Daily energy intake (EI) by chronotype, mealtime regularity, and meal timing.

EI, that is, dietary quantity, did not vary by chronotype, mealtime regularity, breakfast timing, or dinner timing (Figure 2). EI-CV was markedly higher in the irregular mealtime group (*p* < 0.0001); however, slight differences were also observed based on chronotype and dinner timing (Table S2). The evening chronotype demonstrated a higher EI-CV than the intermediate chronotype. We observed a nonlinear association between dinner timing and EI-CV. Specifically, CV levels were significantly increased at both the earlier and later dinner times.

### 3.4. CV of Daily Nutrient Intake by Chrononutritional Classification

**Figure 3:**
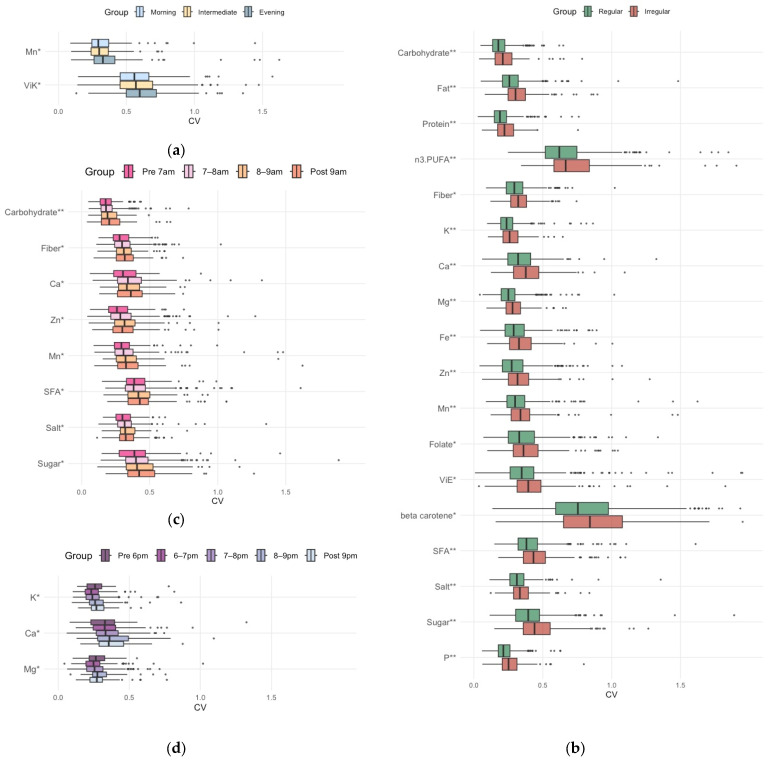
Comparison of the coefficient of variation (CV) of each nutrient between (**a**) chronotype, (**b**) mealtime regularity, (**c**) breakfast timing, and (**d**) dinner timing groups. Boxplots display the median (line) and interquartile range (box). Significance levels are denoted as * *p*-value (a general linear model adjusted for age, BMI, and physical activity) < 0.05, ** *p* < 0.001. Abbreviations: Mn, manganese; ViK, vitamin K; PUFA, polyunsaturated fatty acid; K, potassium; Ca, calcium; Mg, magnesium; ViE, vitamin E; SFA, saturated fatty acid; P, phosphorus; Zn, zinc.

We investigated how day-to-day variability (CV) in daily nutrient intake was associated with chronotype, mealtime regularity, and the timing of breakfast and dinner. Mealtime irregularity was strongly associated with the CV of most nutrient intakes, whereas chronotype and meal timing were associated with the CV of some minerals and dietary fiber (Table S3). Manganese and vitamin K demonstrated a statistically significant increase in CV with a shift toward an evening chronotype (Figure 3a). By contrast, the irregular mealtime group exhibited significantly increased CVs for carbohydrate, fat, protein, *n*-3 polyunsaturated fatty acid (PUFA), dietary fiber, potassium, calcium, magnesium, iron, zinc, manganese, folate, vitamin E, β-carotene, SFA, salt, sugar, and phosphorus (Figure 3b). Sensitivity analyses using alternative thresholds (0.5 h and 1.0 h) also compared the regular mealtime group, the slightly irregular mealtime group, and the irregular mealtime group (Table S4). The CVs for most nutrient intakes were comparable between the regular and slightly irregular mealtime groups but were significantly larger in the irregular mealtime group (Table S4). Other sensitivity analyses were performed by including each individual’s mean snack energy contribution (%E) and the day-to-day variability of snack energy intake (CV), to additionally adjust for proxies of snacking behavior. Consequently, the significantly higher CVs for daily intakes of most nutrients in the irregular mealtime group remained robust (Table S5). Later breakfast timing was associated with increased CVs for carbohydrate, dietary fiber, calcium, zinc, manganese, SFA, salt, and sugars (Figure 3c), whereas later dinner timing was associated with increased CVs for potassium, calcium, and magnesium (Figure 3d).

### 3.5. Ratio to Reference Values of Nutrient Intake by Chrononutritional Classification

**Figure 4:**
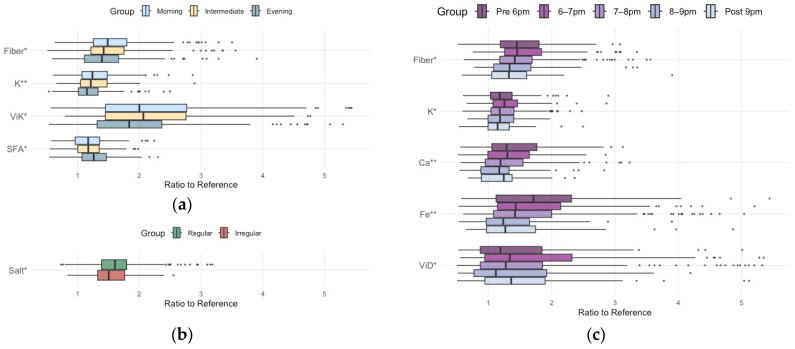
Comparison of ratio to the reference value of each nutrient across (**a**) chronotype, (**b**) mealtime regularity and (**c**) dinner timing groups. Boxplots display the median (line) and interquartile range (box). Significance levels are denoted as * *p*-value (a general linear model adjusted for age, BMI, and physical activity) < 0.05, ** *p* < 0.001. Abbreviations: K, potassium; ViK, vitamin K; SFA, saturated fatty acid; Ca, calcium; Fe, iron; ViD, vitamin D.

Subsequently, we investigated differences in nutrient intake, particularly the ratio to the reference value, based on chronotype, mealtime regularity, breakfast timing, and dinner timing (Figure 4 and Table S6).

Dinner timing and chronotype mainly influenced recommended nutrient intakes relative to reference values, whereas breakfast timing and mealtime regularity showed no statistical association. Regarding dietary fiber, potassium, and vitamin K, the ratio of intake to the reference value was significantly lower in association with a later chronotype (Figure 4a). Moreover, dietary fiber, potassium, calcium, iron, and vitamin D exhibited a significantly reduced intake ratio, especially with a later dinner time (Figure 4c).

Regarding nutrients requiring restriction, such as SFA and salt, all subgroups of chronotype, mealtime regularity, and meal timing showed excessive intake. Furthermore, the evening chronotype exhibited a significantly more pronounced SFA overconsumption (Figure 4a). Although the irregular mealtime group (ratio = 1.5) showed a slightly lower ratio intake of salt than the regular mealtime group (ratio = 1.6), the median values for both groups were more than 1.5-fold the target value (Figure 4b).

### 3.6. Association Between Late Dinner and Lower Diet Quality

The overall adequacy and balance of various nutrients, rather than by the intake of a single nutrient, determine diet quality. Therefore, we assessed daily diet quality using a composite score (see Section 2). The median (Q1–Q3) daily nutritional score was 13.7 (13.3–14.1), with a range of 8.7–15 (Figure S3). As the nutritional score is calculated on the basis of the ratio of nutrient intake to reference values, we investigated whether chronotype and meal timing, which, as noted above, affect these ratios, influence the score.

We initially analyzed whether daily diet quality changes as chronotype shifts from morning to evening or as breakfast/dinner timing becomes later using a generalized additive model with age, BMI, and physical activity as covariates (Table 4). Neither chronotype nor breakfast timing was significantly associated with the nutritional score. By contrast, the nutritional score decreased as dinner time became later (*p* < 0.0351) (Table 4).

**Figure 5:**
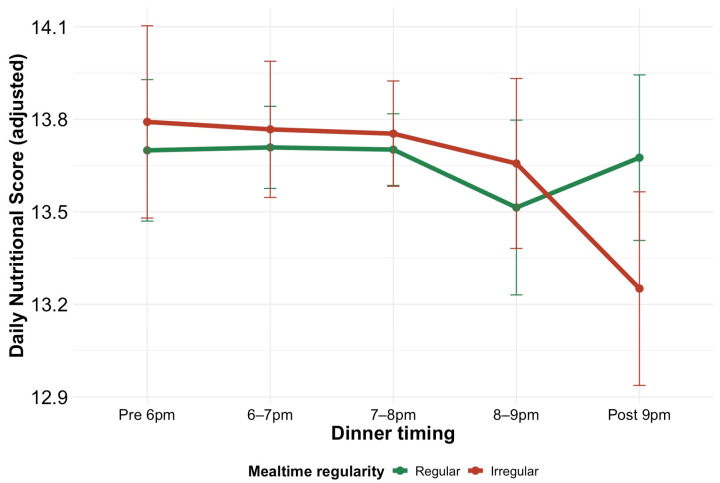
Estimated marginal means of the daily nutritional score across dinner timing, stratified by mealtime regularity. Data are presented as covariate (age, BMI, and physical activity)-adjusted means with 95% confidence intervals.

Subsequently, we examined a potential interaction to explore whether the association between dinner timing and diet quality differs depending on mealtime regularity. The estimated mean daily nutritional scores across dinner timing, adjusted for covariates (age, BMI, and physical activity) for the regular and irregular mealtime groups, are depicted in Figure 5. In the regular mealtime group, scores did not substantially decrease even as dinner timing became later, whereas in the irregular mealtime group, scores sharply declined at later times. Therefore, a marginal interaction was observed between mealtime regularity and dinner timing regarding the nutritional score (*p* for interaction = 0.062), with an apparent difference in the estimated mean scores after 9:00 p.m (13.7 [95% CI: 13.4 to 13.9] for the regular mealtime group vs. 13.3 [12.9 to 13.6] for the irregular mealtime group). The irregular mealtime group demonstrated a significant linear decline in scores with later dinner times (linear trend estimate = −1.19 [−2.11 to −0.27]; *p* for trend = 0.0112), whereas the decline was not significant in the regular mealtime group (linear trend estimate = −0.24 [−0.98 to 0.50]; *p* for trend = 0.5219). Furthermore, sensitivity analyses were performed by including each individual’s mean snack energy contribution (%E) and the day-to-day variability of snack energy intake (CV), to additionally adjust for proxies of snacking behavior. The *p*-value for the interaction between mealtime regularity and dinner timing remained largely unchanged at 0.064, with an apparent difference in the estimated mean scores after 9:00 p.m (13.7 [95% CI: 13.4 to 14.0] for the regular mealtime group vs. 13.3 [13.0 to 13.6] for the irregular mealtime group). The irregular mealtime group demonstrated a significant linear decline in scores with later dinner times (linear trend estimate = −1.24 [−2.14 to −0.34]; *p* for trend = 0.0073), whereas the decline was not significant in the regular mealtime group (linear trend estimate = −0.32 [−1.05 to 0.42]; *p* for trend = 0.3963). These results suggest that the decline in diet quality associated with late dinner timing may be more pronounced in individuals with irregular mealtimes.

## 4. Discussion

This study aimed to elucidate the association between meal timing and mealtime irregularity with diet quality and day-to-day variability (CV) in nutrient intake using real-world longitudinal data. In this study, meal timing refers to participants’ habitual average breakfast and dinner times across the 1-month observation period, whereas mealtime irregularity was quantified using the composite phase deviation (CPD), with higher CPD indicating greater day-to-day mealtime variability. Multiple lines of evidence have indicated that both meal timing and mealtime regularity are associated with the risk of cardiometabolic diseases [4,10,11,26,27,28]. Observational studies have indicated that individuals with evening chronotypes, late meal timings, or irregular eating rhythm tend to demonstrate unhealthy dietary patterns [10,29]. However, the major limitation of these studies is their reliance on self-reported “habitual” mealtimes, capturing only average meal timing. Consequently, the specific impact of mealtime regularity (day-to-day variability) has often been confounded with late meal timing and remains largely unexplored. To bridge this gap, this study investigated how mealtime irregularity is associated with nutrient intake variability and diet quality by leveraging 1 month of comprehensive daily mealtime and intake records.

Regarding mealtime regularity, the results revealed that the irregular mealtime group demonstrated a significantly greater nutrient intake variability than the regular mealtime group; however, mealtime irregularity was not associated with habitual averaged energy and nutrient intake levels. By contrast, regarding habitual meal timing, later dinner timing was associated with lower dietary fiber, potassium, calcium, and iron consumption. Later dinner timing was subsequently associated with lower comprehensive diet quality scores. Intriguingly, this trend appeared more pronounced in the irregular mealtime group, providing suggestive evidence that the association between habitual dinner timing and diet quality may differ depending on mealtime regularity.

Focusing first on habitual meal timing, previous studies have reported that individuals with an evening chronotype show lower adherence to the Mediterranean diet than those with a morning chronotype [3,29]. Additionally, evening types have been demonstrated to have poorer diet quality than morning or intermediate types, characterized by lower plant protein, dietary fiber, fruit, and vegetable consumption [2]. Consistent with these findings, this study revealed that the later chronotype and late dinner timing groups exhibited significantly lower adequacy ratios for dietary fiber and potassium relative to reference values. This finding indicates that the association between eveningness and lower diet quality observed in international studies also applies to our study population, suggesting a common association across various regions.

In contrast to studies on average meal timing, studies focusing on mealtime regularity (day-to-day irregularity) and day-to-day variability (CV) in nutrient intake remain scarce. Of particular note, a recent report by Singh et al. [9] has shown a negative association between gut microbiota diversity and the day-to-day intake variability (CV) of fat, *n*-3 PUFA, potassium, magnesium, vitamin E, beta carotene, SFA, sugar, and phosphorus. In our analysis, greater mealtime irregularity (higher CPD) was associated with higher intake variability for these nutrients. Considering the critical role of gut health in lifestyle-related disease prevention, these findings suggest that dietary health assessments should consider not only habitual nutrient intake levels but also the consistency of intake (i.e., low day-to-day variability). Thus, alongside high average diet quality, maintaining low intake variability may be important, potentially implicating mealtime regularity in this context.

Furthermore, our exploratory analysis provided suggestive evidence of a potential interaction between dinner timing and mealtime regularity concerning diet quality. Although the interaction did not reach conventional statistical significance (*p* for interaction = 0.062), the irregular mealtime group showed a significant linear decrease in nutritional scores with later dinner timing (*p* for trend = 0.0112), whereas no such trend was evident in the regular group (*p* for trend = 0.5219). The divergence in adjusted scores became more apparent at later dinner times (≥9:00 p.m.); however, given the marginal significance of the interaction, these findings should be interpreted cautiously and warrant confirmation in future studies.

The strength of this study lies in its analysis of EI, diet quality, and day-to-day intake variability, conducted after confirming the validity of participants’ reported EI. Furthermore, this study provides a novel approach by evaluating dietary healthfulness through diet quality and consistency of intake.

Our study had several limitations. First, we could not establish causal relationships among chronotype, meal timing, mealtime regularity, and diet quality or intake variability owing to the cross-sectional study design.

Second, although we accounted for height, weight, age, and physical activity, residual confounding is likely in this app-user cohort. We could not perform additional adjustments or sensitivity analyses because data on several important confounders were not collected. These unmeasured factors include socioeconomic status, education level, employment schedules or work patterns (even among non–shift workers), smoking and alcohol consumption, family structure, health-conscious behaviors, stress, mental health and supplement use. It is crucial to emphasize that late or irregular meal patterns may reflect these broader lifestyle factors rather than being isolated behaviors. Because these unmeasured factors may influence both meal timing/regularity and diet quality [10,30,31,32,33,34,35], the observed associations should be interpreted as correlational and hypothesis-generating, rather than as evidence for direct intervention targets. Instead, these findings require confirmation in future prospective and interventional studies to better elucidate the underlying background factors influencing these dietary behaviors.

Third, the study population was restricted to mobile application users who recorded meals almost daily (mean of 30 days during the 1-month period). Such intensive self-monitoring likely selected health-conscious and highly adherent individuals. Consistent with this, the ratios of nutrient intake to reference values were mostly >1.0, indicating healthier profiles than the general population. This “healthy-user” selection may affect not only generalizability but also the magnitude of the observed associations. Specifically, the distributions of outcomes such as diet quality scores may have been shifted upward and compressed, restricting the range and creating ceiling effects that potentially attenuate the observed associations. Conversely, health-conscious users may differ in other health-related behaviors and conditions not fully captured by our adjustment set (age, BMI, and physical activity), which could inflate associations through residual confounding. Furthermore, near-daily logging may reduce dietary measurement error compared with less intensive assessments, potentially making associations appear stronger or more consistent in this cohort. Given these competing methodological factors, the direction and magnitude of selection-related bias are uncertain. Thus, our findings should be interpreted as associations within a highly motivated, health-aware population, and validation in more representative samples is warranted.

Fourth, due to application limitations, we were unable to evaluate the exact timing of snacks. To address this, we performed sensitivity analyses adjusting for proxies of snacking behavior—specifically, the energy contribution from snacks and the day-to-day variability in snack energy intake. Although these proxies cannot directly capture the irregularity of snack timing, the results suggest that our main findings, based on the mealtime irregularity (CPD) of the three main meals, remain robust against the potential underestimation of overall dietary irregularity caused by unobserved snack timing.

Furthermore, we performed a large number of hypothesis tests across multiple nutrients and chrononutritional exposures without adjusting for multiplicity. As these analyses were exploratory, there is an increased risk of Type I errors (false positives). Therefore, the observed associations at the individual nutrient level should be interpreted with appropriate caution and require confirmation in future, focused studies. Despite these limitations, our study offers useful insights. Although genetic and social factors largely determine chronotype, meal timing and regularity are potentially modifiable behaviors. We therefore explored whether mealtime regularity modifies the association between dinner timing and diet quality, while recognizing that these patterns may also reflect broader contextual factors.

## 5. Conclusions

Our high-resolution analysis using mobile application records suggested that lifestyle rhythms—particularly meal timing and regularity—were associated with the adequacy and day-to-day consistency of nutrient intake. From a public health perspective, these exploratory findings imply that recommendations could extend beyond simply advising individuals to ‘eat early.’ However, late or irregular eating patterns may reflect broader lifestyle or socioeconomic constraints rather than being direct targets for intervention. Further prospective and interventional studies are essential to confirm these associations and determine whether modifying meal patterns can effectively improve nutritional outcomes.

## Figures and Tables

**Table 1:** List of nutrients used to assess day-to-day intake variability in this study.

Nutrient	CV Correlates with Gut Alpha-Diversity ^†^
Carbohydrate	
Fat	yes
Protein	
*n*-3 polyunsaturated fatty acids (PUFA)	yes
Dietary fiber	
Potassium (K)	yes
Calcium (Ca)	
Magnesium (Mg)	yes
Iron (Fe)	
Zinc (Zn)	
Manganese (Mn)	
Folate	
Vitamin C (ViC)	
Vitamin A (ViA)	
Vitamin D (ViD)	
Vitamin E (ViE)	yes (Oil nuts)
Vitamin K (ViK)	
Beta-carotene	yes
Saturated fatty acids (SFA)	yes
Sodium (Salt Equivalent)	
Sugar ^§^	yes
Phophorus	yes

^†^ Reported in ref. [9]. ^§^ Sugar is the sum of glucose, fructose, galactose, sucrose, maltose, lactose, and trehalose. Abbreviations: CV, coefficient of variation.

**Table 2:** List of nutrients for evaluating adherence to the Dietary Reference Intake for Japanese and their reference values used in this study.

Nutrient	Reference Value
Adequacy components (recommended nutrients)	
Protein	DG ^†^
*n*-3 polyunsaturated fatty acids (PUFA)	AI
Dietary fiber	DG
Potassium (K)	DG
Calcium (Ca)	RDA
Magnesium (Mg)	RDA
Iron (Fe)	RDA
Zinc (Zn)	RDA
Manganese (Mn)	AI
Folate	RDA
Vitamin C (ViC)	RDA
Vitamin A (ViA)	RDA
Vitamin D (ViD)	AI
Vitamin E (ViE)	AI
Vitamin K (ViK)	AI
Moderation components (nutrients to be limited)	
Saturated fatty acids (SFA)	DG
Sodium (Salt Equivalent)	DG

^†^ The lower limit of the tentative dietary goal, DG (13% of the energy intake). Abbreviations: RDA, recommended dietary allowance; AI, adequate intake; DG, tentative dietary goal for preventing lifestyle-related diseases.

**Table 3:** Participant characteristics.

Nutrient	Regular Mealtime Group [*n* = 531]		Irregular Mealtime Group [*n* = 209]	
	Mean or *n*	SD or %	Mean or *n*	SD or %
Age (years old) *	42.1	11.2	40.0	10.8
Height (cm)	158.3	5.4	158.4	5.1
Weight (kg)	55.5	10.5	55.7	10.2
BMI (kg/m^2^)	22.2	4.0	22.2	3.8
Wake time (hh:mm) on workdays **	6:16	1:09	6:41	1:25
Wake time (hh:mm) on free days **	7:11	1:31	7:51	1:36
Sleep onset time (hh:mm) on workdays **	23:34	1:10	23:58	1:13
Sleep onset time (hh:mm) on free days **	23:48	1:13	24:11	1:19
Sleep duration (hh:mm) on workdays	6:42	0:58	6:43	1:10
Sleep duration (hh:mm) on free days *	7:22	1:10	7:39	1:17
MSFsc (hh:mm) **	3:14	1:08	3:40	1:17
Chronotype **				
Morning type	164	30.9	46	22.0
Intermediate type	199	37.5	63	30.1
Evening type	168	31.6	100	47.8
Physical activity (MET-h/week)	32.2	28.7	34.8	34.1
Breakfast time (hh:mm) **	7:42	1:12	8:21	1:17
Lunch time (hh:mm) **	12:29	0:49	13:03	1:04
Dinner time (hh:mm) **	19:01	1:07	19:25	1:21
Breakfast time CPD (h) **	0.13	0.25	1.20	0.89
Lunch time CPD (h) **	0.12	0.25	1.14	0.79
Dinner time CPD (h) **	0.10	0.23	1.07	0.76

Significance levels are denoted as * *p*-value (*t*-test or Pearson’s chi-square test) < 0.05, ** *p* < 0.001. *n*, Sample size; Abbreviations: SD, standard deviation; BMI, body mass index; MSFsc, sleep-corrected midpoint on free days; MET, metabolic equivalent of task; CPD, composite phase deviation.

**Table 4:** Regression analyses of chronotype and meal timing associated with the nutritional score.

**(a) Chronotype**			
**Explanatory Variable**	**Coefficient (β)**	**95% CI**	***p*** **-Value**
Chronotype (ordinal variable)	−0.035	−0.104, 0.034	0.3184
Age (years)	0.008	0.003, 0.013	0.0025
BMI (kg/m^2^)	−0.008	−0.022, 0.005	0.2325
PA (MET-hour/week)	0.002	0.001, 0.004	0.0117
**(b) Breakfast Timing**			
**Explanatory Variable**	**Coefficient (β)**	**95% CI**	***p*** **-Value**
Breakfast timing (ordinal variable)	0.026	−0.028, 0.081	0.3447
Age (years)	0.008	0.004, 0.013	0.0007
BMI (kg/m^2^)	−0.010	−0.023, 0.004	0.1578
PA (MET-hour/week)	0.002	0.000, 0.004	0.0133
**(c) Dinner Timing**			
**Explanatory Variable**	**Coefficient (β)**	**95% CI**	***p*** **-Value**
Dinner timing (ordinal variable)	−0.059	−0.113, −0.004	0.0351
Age (years)	0.008	0.003, 0.013	0.0009
BMI (kg/m^2^)	−0.009	−0.022, 0.005	0.2062
PA (MET-hour/week)	0.002	0.001, 0.004	0.0086

Abbreviations: BMI, body mass index; PA, physical activity; MET, metabolic equivalent of task; CI, confidence interval.

## Data Availability

The data used in this study, purchased from asken, are the property of the company and will not be released to the public.

## Supplementary Materials

The following supporting information can be downloaded at: https://www.mdpi.com/article/10.3390/nu18060957/s1, Table S1. Sensitivity analysis using alternative cutoffs for mealtime regularity (CPD): participant characteristics. Table S2. Coefficient of variation (CV) of energy intake by chronotype, mealtime regularity, breakfast timing, and dinner timing; Table S3. The day-to-day variability (CV) in daily nutrient intake by chronotype, mealtime regularity, breakfast timing, and dinner timing; Table S4. Sensitivity analysis using alternative cutoffs for mealtime regularity (CPD): the day-to-day variability (CV) in daily nutrient intake; Table S5. Sensitivity analysis: additional adjustment for mean snack energy contribution (%E) and the day-to-day variability of snack energy intake (CV); Table S6. The ratio to reference daily value by chronotype, mealtime regularity, breakfast timing, and dinner timing; Figure S1. Conceptual illustration of the Composite Phase Deviation (CPD) and representative 1-month mealtime patterns; Figure S2. Weight change by EI:BMR ratio group; Figure S3. Nutritional score distribution.

## Abbreviations

The following abbreviations are used in this manuscript:

CV	Coefficient of Variation
DRIs-J	Dietary Reference Intakes for Japanese
SFA	Saturated Fatty Acids
EI	Energy Intake
BMI	Body Mass Index
MSFsc	Sleep-corrected midpoint on free days
CPD	Composite Phase Deviation
STFC	Standard Tables of Food Composition
AI	Adequate Intake
DG	Dietary Goal for preventing lifestyle-related diseases
PUFA	Poly Unsaturated Fatty Acids
NRF	Nutrient-Rich Food Index
EE	Energy Expenditure
BMR	Basal Metabolic Rate
FAO	Food and Agriculture Organization
WHO	World Health Organization
UNU	United Nations University
CI	Confidence Interval
PAL	Physical Activity Level
MET	Metabolic Equivalent of Task

## References

[1] Leech R.M., Timperio A., Livingstone K.M., Worsley A., McNaughton S.A. (2017). Temporal Eating Patterns: Associations with Nutrient Intakes, Diet Quality, and Measures of Adiposity. Am. J. Clin. Nutr..

[2] Zuraikat F.M., St-Onge M.-P., Makarem N., Boege H.L., Xi H., Aggarwal B. (2021). Evening Chronotype Is Associated with Poorer Habitual Diet in US Women, with Dietary Energy Density Mediating a Relation of Chronotype with Cardiovascular Health. J. Nutr..

[3] Godos J., Castellano S., Ferri R., Caraci F., Lanza G., Scazzina F., Alanazi A.M., Marx W., Galvano F., Grosso G. (2023). Mediterranean Diet and Chronotype: Data from Italian Adults and Systematic Review of Observational Studies. Exp. Gerontol..

[4] Teixeira G.P., da Cunha N.B., Azeredo C.M., Rinaldi A.E.M., Crispim C.A. (2023). Eating Time Variation from Weekdays to Weekends and Its Association with Dietary Intake and BMI in Different Chronotypes: Findings from National Health and Nutrition Examination Survey (NHANES) 2017–2018. Br. J. Nutr..

[5] Flanagan A., Bechtold D.A., Pot G.K., Johnston J.D. (2021). Chrono-nutrition: From Molecular and Neuronal Mechanisms to Human Epidemiology and Timed Feeding Patterns. J. Neurochem..

[6] Mistlberger R.E. (2020). Food as Circadian Time Cue for Appetitive Behavior. F1000Research.

[7] Barrett E.M., Afrin H., Rayner M., Pettigrew S., Gaines A., Maganja D., Jones A., Mozaffarian D., Beck E.J., Neal B. (2024). Criterion Validation of Nutrient Profiling Systems: A Systematic Review and Meta-Analysis. Am. J. Clin. Nutr..

[8] Wirt A., Collins C.E. (2009). Diet Quality—What Is It and Does It Matter?. Public Health Nutr..

[9] Singh R., McDonald D., Hernandez A.R., Song S.J., Bartko A., Knight R., Salathé M. (2025). Temporal Nutrition Analysis Associates Dietary Regularity and Quality with Gut Microbiome Diversity: Insights from the Food & You Digital Cohort. Nat. Commun..

[10] Sierra-Johnson J., Undén A., Linestrand M., Rosell M., Sjogren P., Kolak M., De Faire U., Fisher R.M., Hellénius M. (2008). Eating Meals Irregularly: A Novel Environmental Risk Factor for the Metabolic Syndrome. Obesity.

[11] Suyoto P.S., Pamungkas N.P., De Vries J.H., Feskens E.J. (2024). Associations between Variability in Between- and Within-Day Dietary Intake with Adiposity and Glucose Homeostasis in Adults: A Systematic Review. Adv. Nutr..

[12] Sato N., Terasaki H., Tahara Y., Michie M., Umezawa A., Shibata S. (2025). Day-to-Day Variability in Meal Timing and Its Association with Body Mass Index: A Study Using Data from a Japanese Food-Logging Mobile Application. Nutrients.

[13] Seol J., Iwagami M., Kayamare M.C.T., Yanagisawa M. (2025). Relationship Among Macronutrients, Dietary Components, and Objective Sleep Variables Measured by Smartphone Apps: Real-World Cross-Sectional Study. J. Med. Internet Res..

[14] Matsuzaki E., Michie M., Kawabata T. (2017). Validity of Nutrient Intakes Derived from an Internet Website Dish-Based Dietary Record for Self-Management of Weight among Japanese Women. Nutrients.

[15] Nitta L., Tahara Y., Shinto T., Makino S., Kuwahara M., Tada A., Abe N., Michie M., Shibata S. (2023). Association of Eating Pattern, Chronotype, and Social Jetlag: A Cross-Sectional Study Using Data Accumulated in a Japanese Food-Logging Mobile Health Application. Nutrients.

[16] Imamura M., Sasaki H., Shinto T., Tahara Y., Makino S., Kuwahara M., Tada A., Abe N., Michie M., Shibata S. (2022). Association Between Na, K, and Lipid Intake in Each Meal and Blood Pressure. Front. Nutr..

[17] Craig C.L., Marshall A.L., Sjöström M., Bauman A.E., Booth M.L., Ainsworth B.E., Pratt M., Ekelund U., Yngve A., Sallis J.F. (2003). International Physical Activity Questionnaire: 12-Country Reliability and Validity. Med. Sci. Sports Exerc..

[18] Kitamura S., Hida A., Aritake S., Higuchi S., Enomoto M., Kato M., Vetter C., Roenneberg T., Mishima K. (2014). Validity of the Japanese Version of the Munich ChronoType Questionnaire. Chronobiol. Int..

[19] McHill A.W., Hilditch C.J., Fischer D., Czeisler C.A., Garaulet M., Scheer F.A.J.L., Klerman E.B. (2020). Stability of the Timing of Food Intake at Daily and Monthly Timescales in Young Adults. Sci. Rep..

[20] Ministry of Education, Culture, Sports, Science and Technology-Japan (MEXT) Standard Tables of Food Composition in Japan 2020 (8th Edition). https://www.mext.go.jp/a_menu/syokuhinseibun/mext_01110.html.

[21] Ministry of Health, Labour and Welfare (2025). Dietary Reference Intakes for Japanese. https://www.mhlw.go.jp/stf/newpage_44138.html.

[22] Drewnowski A. (2010). The Nutrient Rich Foods Index Helps to Identify Healthy, Affordable Foods. Am. J. Clin. Nutr..

[23] Black A. (2000). Critical Evaluation of Energy Intake Using the Goldberg Cut-off for Energy Intake: Basal Metabolic Rate. A Practical Guide to Its Calculation, Use and Limitations. Int. J. Obes..

[24] Ganpule A.A., Tanaka S., Ishikawa-Takata K., Tabata I. (2007). Interindividual Variability in Sleeping Metabolic Rate in Japanese Subjects. Eur. J. Clin. Nutr..

[25] Human Energy Requirements. https://www.fao.org/4/y5686e/y5686e00.htm.

[26] Gill S., Panda S. (2015). A Smartphone App Reveals Erratic Diurnal Eating Patterns in Humans That Can Be Modulated for Health Benefits. Cell Metab..

[27] Manoogian E.N.C., Chaix A., Panda S. (2019). When to Eat: The Importance of Eating Patterns in Health and Disease. J. Biol. Rhythm..

[28] McHill A.W., Phillips A.J., Czeisler C.A., Keating L., Yee K., Barger L.K., Garaulet M., Scheer F.A., Klerman E.B. (2017). Later Circadian Timing of Food Intake Is Associated with Increased Body Fat. Am. J. Clin. Nutr..

[29] Muscogiuri G., Barrea L., Aprano S., Framondi L., Di Matteo R., Laudisio D., Pugliese G., Savastano S., Colao A., On Behalf of The Opera Prevention Project (2020). Chronotype and Adherence to the Mediterranean Diet in Obesity: Results from the Opera Prevention Project. Nutrients.

[30] Ciobanu D., Porojan M., Bala C., Zah A.M., Oroian I., Roman G., Rusu A. (2024). Lifestyle Factors, Dietary Patterns, and Social Determinants of Social and Eating Jetlag: A Cross-Sectional Survey. Chronobiol. Int..

[31] Park J., Yeo Y., Yoo J.H. (2022). Dietary Intake and Nutritional Status in Young and Middle-Aged Adults According to the Meal Frequency from the Korea National Health and Nutritional Survey. Korean J. Fam. Med..

[32] Kabalı S., Çelik M.N., Öner N. (2025). Associations of Nutrition Education with Diet Quality Indexes and Chronotype: A Cross-Sectional Study. Int. J. Environ. Health Res..

[33] Hayashida T., Shimura A., Higashiyama M., Fujimura Y., Ono K., Inoue T. (2021). Psychosomatic Stress Responses and Sleep Disturbance Mediate the Effects of Irregular Mealtimes on Presenteeism. Neuropsychiatr. Dis. Treat..

[34] Huseinovic E., Winkvist A., Freisling H., Slimani N., Boeing H., Buckland G., Schwingshackl L., Olsen A., Tjønneland A., Stepien M. (2019). Timing of Eating across Ten European Countries—Results from the European Prospective Investigation into Cancer and Nutrition (EPIC) Calibration Study. Public Health Nutr..

[35] Allen M.S., Mishra M., Tashjian S.M., Laborde S. (2025). Linking Big Five Personality Traits to Components of Diet: A Meta-Analytic Review. J. Personal. Soc. Psychol..

