# Supplementary material for: Associations of Temporal Eating Patterns with Nutrient Intake Variability and Diet Quality Among Japanese Female Mobile Application Users
Source: Nutrients. 2026 Mar 18;18(6):957. doi: 10.3390/nu18060957 (PMC13028760; doi:10.3390/nu18060957)
Supplement: Supplementary file 1 [file nutrients-18-00957-s001.zip › Supplementary Materials 20260312.pdf]

Supplementary Materials for Umezawa et al. "Associations of Temporal Eating Patterns with Nutrient Intake Variability and Diet Quality among Japanese Female Mobile Application Users".

Table S1. Sensitivity analysis using alternative cutoffs for mealtime regularity (CPD): participant characteristics

Table S2. Coefficient of variation (CV) of energy intake by chronotype, mealtime regularity, breakfast timing, and dinner timing

Table S3. The day-to-day variability (CV) in daily nutrient intake by chronotype, mealtime regularity, breakfast timing, and dinner timing

Table S4. Sensitivity analysis using alternative cutoffs for mealtime regularity (CPD): the day-to-day variability (CV) in daily nutrient intake

Table S5. Sensitivity analysis: additional adjustment for mean snack energy contribution (%E) and the day-to-day variability of snack energy intake (CV)

Table S6. The ratio to reference daily value by chronotype, mealtime regularity, breakfast timing, and dinner timing

Figure S1. Conceptual illustration of the Composite Phase Deviation (CPD) and representative 1-month mealtime patterns

Figure S2. Weight change by EI:BMR ratio group

Figure S3. Nutritional score distribution

Table S1. Sensitivity analysis using alternative cutoffs for mealtime regularity (CPD): participant characteristics

	Regular mealtime group (n = 420)		Slightly Irregular mealtime group (n = 111)		Irregular mealtime group (n = 209)	
Variable	Mean or n	SD or %	Mean or n	SD or %	Mean or n	SD or %
Age (years old)	42.1	11.2	41.9	11.3	40.0	10.8
Height (cm)	158.4	5.4	157.6	5.6	158.4	5.1
Weight (kg)	55.2	9.9	56.9	12.5	55.7	10.2
BMI (kg/m^2)	22.0	3.8	22.9	4.8	22.2	3.8
Wake time (hh:mm) on workdays*	6:17	1:11 [a]	6:12	0:59 [a]	6:41	1:24 [b]
Wake time (hh:mm) on free days**	7:13	1:34 [a]	7:01	1:17 [a]	7:50	1:36 [b]
Sleep onset time (hh:mm) on workdays**	23:34	1:10 [a]	23:33	1:08 [a]	23:57	1:13 [b]
Sleep onset time (hh:mm) on free days**	23:49	1:12 [a]	23:42	1:11 [a]	0:11	1:18 [b]
Sleep duration (hh:mm) on workdays	6:42	0:56	6:38	1:02	6:43	1:10
Sleep duration (hh:mm) on free days*	7:23	1:11 [a]	7:18	1:06 [ab]	7:39	1:17 [b]
MSFsc (hh:mm)**	3:15	1:10 [a]	3:06	1:02 [a]	3:39	1:17 [b]
Chronotype **						
Morning type	129	30.7 [a]	35	31.5 [a]	46	22.0 [b]
Intermediate type	154	36.7 [a]	45	40.5 [b]	63	30.1 [c]
Evening type	137	32.6 [a]	31	27.9 [b]	100	47.8 [c]
Physical activity (MET-h/week)	32.6	30.3	30.4	21.7	34.8	34.1
Breakfast time (hh:mm)**	7:41	1:15 [a]	7:43	1:00 [a]	8:21	1:16 [b]
Lunch time (hh:mm)**	12:25	0:50 [a]	12:39	0:41 [b]	13:03	1:04 [c]
Dinner time (hh:mm)**	19:00	1:05 [a]	19:04	1:11 [a]	19:24	1:21 [b]
Breakfast time CPD (h)**	0.03	0.10 [a]	0.48	0.31 [b]	1.20	0.89 [c]
Lunch time CPD (h)**	0.02	0.08 [a]	0.50	0.29 [b]	1.14	0.79 [c]
Dinner time CPD (h)**	0.02	0.07 [a]	0.41	0.32 [b]	1.07	0.76 [c]

Significance levels are denoted as * p-value (Kruskal-Wallis test or Pearson's chi-square test) < 0.05, ** < 0.001.
When the Kruskal-Wallis test yielded significant results, a post hoc Bonferroni-corrected Dunn's test was performed.
Values with different superscript letters indicate significant differences ($p < 0.05$),
whereas those sharing the same letter are not significantly different.

Table S2. Coefficient of variation (CV) of energy intake by chronotype, mealtime regularity, breakfast timing, and dinner timing.

(a) Chronotype

	Median (Q1-Q3)		
Coefficient of Variation	**Morning**	**Intermediate**	**Evening**
Energy intake CV [a]	0.137 (0.096-0.187)	0.132 (0.100-0.172)	0.147 (0.114-0.192)

(b) Mealtime regularity

	Median (Q1-Q3)	
Coefficient of Variation	**Regular**	**Irregular**
Energy intake CV [b]	0.131 (0.096-0.172)	0.166 (0.123-0.231)

(c) Breakfast timing

	Median (Q1-Q3)			
Coefficient of Variation	**Pre7am**	**7-8am**	**8-9am**	**Post9am**
Energy intake CV	0.144 (0.095-0.177)	0.132 (0.107-0.176)	0.153 (0.108-0.212)	0.140 (0.107-0.210)

(d) Dinner timing

	Median (Q1-Q3)				
Coefficient of Variation	**Pre6pm**	**6-7pm**	**7-8pm**	**8-9pm**	**Post9pm**
Energy intake CV [c]	0.156 (0.115-0.220)	0.136 (0.096-0.178)	0.135 (0.102-0.176)	0.158 (0.126-0.223)	0.145 (0.109-0.225)

[a] p-value (Kruskal-Wallis test) = 0.014. Evening group was significantly higher than Intermediate group (post hoc Dunn's test p = 0.0082)
[b] p-value (Mann-Whitney U test) < 0.00001. *p*-value (a general linear model adjusted for age, BMI, and physical activity) < 0.00001 (increasing trend).
[c] *p*-value (Quadratic model adjusted for age and BMI) < 0.005 (non-linear association).

Table S3. The day-to-day variation (CV) by Chronotype, Mealtime Regularity, Breakfast Timing, and Dinner Timing.

(a) Chronotype

Nutrients	Morning	Intermediate	Evening
Carbohydrate	0.182 (0.139-0.232)	0.177 (0.138-0.226)	0.197 (0.147-0.257)
Fat	0.267 (0.205-0.336)	0.255 (0.211-0.317)	0.281 (0.232-0.350)
Protein	0.196 (0.149-0.256)	0.190 (0.150-0.235)	0.208 (0.162-0.264)
n-3 polyunsaturated fatty acids (PUFA)	0.613 (0.512-0.727)	0.634 (0.527-0.781)	0.666 (0.576-0.796)
Dietary fiber	0.280 (0.232-0.351)	0.283 (0.239-0.337)	0.320 (0.261-0.382)
Potassium	0.232 (0.191-0.276)	0.233 (0.198-0.284)	0.259 (0.211-0.309)
Calcium	0.315 (0.243-0.404)	0.338 (0.261-0.415)	0.350 (0.269-0.457)
Magnesium	0.251 (0.199-0.298)	0.257 (0.206-0.306)	0.268 (0.216-0.328)
Iron	0.287 (0.223-0.376)	0.290 (0.228-0.362)	0.314 (0.247-0.387)
Zinc	0.269 (0.193-0.351)	0.285 (0.221-0.360)	0.309 (0.230-0.382)
Manganese *	0.293 (0.246-0.370)	0.300 (0.240-0.366)	0.327 (0.262-0.415)
Folate	0.318 (0.249-0.439)	0.334 (0.257-0.445)	0.355 (0.275-0.453)
Vitamin C	0.475 (0.319-0.665)	0.481 (0.368-0.728)	0.508 (0.356-0.720)
Vitamin A	0.535 (0.384-0.876)	0.535 (0.386-0.784)	0.549 (0.383-0.819)
Vitamin D	0.765 (0.555-1.006)	0.768 (0.605-1.047)	0.783 (0.568-1.000)
Vitamin E	0.360 (0.267-0.452)	0.357 (0.277-0.437)	0.362 (0.283-0.486)
Vitamin K *	0.558 (0.454-0.664)	0.569 (0.453-0.693)	0.597 (0.499-0.720)
Beta carotene	0.767 (0.595-0.989)	0.776 (0.603-0.993)	0.798 (0.624-1.037)
Saturated fatty acids	0.392 (0.322-0.472)	0.383 (0.323-0.466)	0.414 (0.346-0.490)
Sodium (Salt)	0.312 (0.265-0.372)	0.310 (0.262-0.352)	0.327 (0.280-0.390)
Sugar	0.393 (0.294-0.471)	0.400 (0.318-0.482)	0.422 (0.340-0.528)
Phosphorus	0.215 (0.172-0.280)	0.218 (0.184-0.263)	0.237 (0.188-0.289)

(b) Mealtime regularity

Nutrients	Regular	Irregular
Carbohydrate **	0.177 (0.136-0.224)	0.210 (0.154-0.276)
Fat **	0.258 (0.208-0.322)	0.302 (0.248-0.374)
Protein **	0.189 (0.149-0.236)	0.222 (0.172-0.288)
n-3 polyunsaturated fatty acids (PUFA)**	0.620 (0.518-0.749)	0.666 (0.581-0.838)
Dietary fiber *	0.293 (0.235-0.355)	0.322 (0.267-0.382)
Calcium **	0.321 (0.246-0.412)	0.377 (0.288-0.471)
Magnesium **	0.250 (0.198-0.298)	0.280 (0.233-0.338)
Potassium **	0.237 (0.195-0.282)	0.259 (0.211-0.320)
Iron **	0.289 (0.226-0.365)	0.327 (0.259-0.416)
Zinc **	0.274 (0.207-0.356)	0.317 (0.251-0.397)
Manganese **	0.299 (0.240-0.369)	0.338 (0.271-0.405)
Folate *	0.329 (0.250-0.439)	0.360 (0.285-0.466)
Vitamin C	0.484 (0.331-0.703)	0.501 (0.375-0.742)
Vitamin A	0.535 (0.383-0.808)	0.560 (0.386-0.840)
Vitamin D	0.773 (0.575-1.036)	0.768 (0.581-0.971)
Vitamin E *	0.348 (0.264-0.439)	0.395 (0.314-0.488)
Vitamin K	0.569 (0.465-0.694)	0.593 (0.473-0.719)
Beta carotene *	0.755 (0.594-0.978)	0.843 (0.652-1.080)
Saturated fatty acids **	0.381 (0.321-0.461)	0.432 (0.359-0.519)
Sodium (Salt) **	0.312 (0.261-0.363)	0.334 (0.288-0.395)
Sugar **	0.394 (0.303-0.476)	0.440 (0.358-0.554)
Phosphorus **	0.214 (0.177-0.262)	0.251 (0.206-0.313)

Continued

Continued

(c) Breakfast timing

Nutrients	Pre7am	7-8am	8-9am	Post9am
Carbohydrate**	0.176 (0.136-0.217)	0.179 (0.142-0.224)	0.190 (0.146-0.258)	0.202 (0.144-0.280)
Fat	0.257 (0.211-0.328)	0.262 (0.213-0.326)	0.284 (0.220-0.354)	0.275 (0.219-0.368)
Protein	0.205 (0.155-0.252)	0.189 (0.150-0.244)	0.218 (0.167-0.265)	0.199 (0.152-0.254)
n-3 polyunsaturated fatty acids (PUFA)	0.605 (0.502-0.712)	0.644 (0.546-0.783)	0.643 (0.557-0.762)	0.680 (0.528-0.837)
Dietary fiber *	0.279 (0.232-0.346)	0.297 (0.242-0.355)	0.310 (0.256-0.364)	0.316 (0.255-0.379)
Potassium	0.235 (0.183-0.273)	0.243 (0.199-0.292)	0.250 (0.206-0.303)	0.248 (0.204-0.293)
Calcium *	0.303 (0.235-0.399)	0.339 (0.264-0.439)	0.332 (0.270-0.426)	0.361 (0.263-0.444)
Magnesium	0.246 (0.195-0.293)	0.258 (0.196-0.305)	0.268 (0.225-0.332)	0.257 (0.210-0.313)
Iron	0.282 (0.227-0.359)	0.293 (0.229-0.376)	0.316 (0.248-0.394)	0.311 (0.241-0.382)
Zinc *	0.259 (0.198-0.338)	0.284 (0.214-0.363)	0.315 (0.242-0.392)	0.298 (0.229-0.377)
Manganese *	0.292 (0.240-0.351)	0.307 (0.245-0.377)	0.322 (0.254-0.401)	0.326 (0.265-0.414)
Folate	0.324 (0.242-0.473)	0.333 (0.248-0.429)	0.358 (0.284-0.467)	0.348 (0.266-0.447)
Vitamin C	0.457 (0.311-0.661)	0.491 (0.339-0.724)	0.519 (0.369-0.768)	0.491 (0.362-0.699)
Vitamin A	0.559 (0.405-0.797)	0.520 (0.374-0.777)	0.564 (0.415-0.925)	0.533 (0.372-0.827)
Vitamin D	0.792 (0.593-1.060)	0.763 (0.565-0.991)	0.783 (0.579-1.045)	0.761 (0.577-1.058)
Vitamin E	0.358 (0.270-0.434)	0.359 (0.267-0.450)	0.365 (0.296-0.474)	0.356 (0.281-0.456)
Vitamin K	0.546 (0.433-0.647)	0.587 (0.469-0.718)	0.579 (0.475-0.697)	0.588 (0.471-0.704)
Beta carotene	0.731 (0.558-0.965)	0.824 (0.625-1.046)	0.797 (0.633-1.004)	0.741 (0.585-0.954)
Saturated fatty acids *	0.386 (0.329-0.464)	0.381 (0.321-0.469)	0.417 (0.340-0.501)	0.427 (0.341-0.490)
Sodium (Salt) *	0.299 (0.251-0.358)	0.315 (0.265-0.365)	0.320 (0.284-0.391)	0.324 (0.279-0.382)
Sugar *	0.387 (0.275-0.469)	0.399 (0.323-0.487)	0.407 (0.327-0.524)	0.422 (0.339-0.536)
Phosphorus	0.214 (0.171-0.270)	0.218 (0.182-0.276)	0.242 (0.192-0.289)	0.224 (0.187-0.273)

(d) Dinner timing

Nutrients	Pre6pm	6-7pm	7-8pm	8-9pm	Post9pm
Carbohydrate	0.198 (0.153-0.255)	0.185 (0.139-0.235)	0.177 (0.136-0.234)	0.204 (0.160-0.273)	0.211 (0.150-0.325)
Fat	0.295 (0.221-0.376)	0.264 (0.214-0.344)	0.265 (0.212-0.327)	0.288 (0.239-0.350)	0.252 (0.215-0.363)
Protein	0.215 (0.168-0.260)	0.186 (0.146-0.255)	0.198 (0.155-0.245)	0.208 (0.152-0.272)	0.211 (0.180-0.265)
n-3 polyunsaturated fatty acids (PUFA)	0.621 (0.523-0.763)	0.624 (0.534-0.783)	0.635 (0.539-0.747)	0.654 (0.524-0.857)	0.682 (0.599-0.781)
Dietary fiber	0.312 (0.258-0.365)	0.296 (0.238-0.347)	0.289 (0.242-0.355)	0.327 (0.273-0.418)	0.333 (0.265-0.385)
Potassium *	0.259 (0.202-0.306)	0.232 (0.189-0.282)	0.240 (0.195-0.287)	0.259 (0.219-0.316)	0.268 (0.233-0.321)
Calcium *	0.329 (0.230-0.396)	0.327 (0.246-0.404)	0.333 (0.268-0.421)	0.360 (0.278-0.493)	0.355 (0.287-0.460)
Magnesium *	0.264 (0.217-0.327)	0.248 (0.195-0.293)	0.255 (0.200-0.315)	0.275 (0.242-0.339)	0.272 (0.223-0.311)
Iron	0.313 (0.233-0.378)	0.295 (0.221-0.372)	0.293 (0.231-0.369)	0.322 (0.258-0.395)	0.327 (0.266-0.397)
Zinc	0.275 (0.187-0.413)	0.276 (0.219-0.351)	0.283 (0.217-0.364)	0.318 (0.263-0.364)	0.328 (0.236-0.407)
Manganese	0.331 (0.257-0.448)	0.302 (0.242-0.377)	0.298 (0.240-0.370)	0.346 (0.295-0.403)	0.327 (0.270-0.387)
Folate	0.344 (0.258-0.505)	0.331 (0.251-0.427)	0.340 (0.250-0.438)	0.382 (0.276-0.477)	0.371 (0.267-0.480)
Vitamin C	0.545 (0.387-0.828)	0.469 (0.319-0.641)	0.484 (0.351-0.742)	0.555 (0.375-0.837)	0.501 (0.371-0.653)
Vitamin A	0.539 (0.393-0.935)	0.545 (0.377-0.769)	0.528 (0.379-0.792)	0.568 (0.421-0.861)	0.516 (0.391-1.038)
Vitamin D	0.667 (0.542-0.943)	0.764 (0.556-1.059)	0.778 (0.580-1.002)	0.819 (0.607-1.093)	0.809 (0.665-0.973)
Vitamin E	0.362 (0.279-0.456)	0.345 (0.270-0.445)	0.365 (0.270-0.455)	0.371 (0.333-0.463)	0.367 (0.298-0.477)
Vitamin K	0.569 (0.454-0.688)	0.575 (0.463-0.708)	0.568 (0.463-0.691)	0.659 (0.509-0.754)	0.599 (0.472-0.714)
Beta carotene	0.886 (0.656-1.035)	0.790 (0.585-1.001)	0.753 (0.604-0.961)	0.889 (0.679-1.122)	0.793 (0.625-1.023)
Saturated fatty acids	0.424 (0.339-0.543)	0.397 (0.323-0.496)	0.399 (0.328-0.463)	0.387 (0.341-0.478)	0.375 (0.324-0.474)
Sodium (Salt)	0.342 (0.290-0.408)	0.307 (0.263-0.353)	0.315 (0.265-0.370)	0.335 (0.298-0.410)	0.317 (0.262-0.375)
Sugar	0.398 (0.303-0.486)	0.398 (0.317-0.489)	0.403 (0.318-0.484)	0.414 (0.349-0.528)	0.453 (0.349-0.555)
Phosphorus	0.227 (0.186-0.295)	0.218 (0.183-0.270)	0.218 (0.177-0.272)	0.243 (0.209-0.295)	0.241 (0.188-0.301)

When significant differences were observed between groups by the Kruskal-Wallis test (or Mann-Whitney U test), association was further analyzed using a generalized linear model adjusted for age, BMI and physical activity. *, $p < 0.05$; **, $p < 0.001$.

Table S4. Sensitivity analysis using alternative cutoffs for mealtime regularity (CPD): the day-to-day variability (CV) in daily nutrient intake

Nutrients	Regular	Slightly Irregular	Irregular
Carbohydrate **	0.178 (0.136-0.224)[a]	0.177 (0.137-0.234)[a]	0.210 (0.154-0.276)[b]
Fat **	0.255 (0.205-0.321)[a]	0.259 (0.217-0.327)[a]	0.302 (0.248-0.374)[b]
Protein **	0.188 (0.149-0.234)[a]	0.195 (0.147-0.244)[a]	0.222 (0.172-0.288)[b]
n-3 polyunsaturated fatty acids (PUFA)**	0.621 (0.518-0.740)[a]	0.619 (0.517-0.778)[a]	0.666 (0.581-0.838)[b]
Dietary fiber *	0.293 (0.235-0.359)[a]	0.294 (0.235-0.339)[a]	0.322 (0.267-0.382)[b]
Calcium *	0.237 (0.195-0.282)[a]	0.236 (0.192-0.286)[a]	0.259 (0.211-0.320)[b]
Magnesium **	0.317 (0.245-0.413)[a]	0.332 (0.257-0.406)[a]	0.377 (0.288-0.471)[b]
Potassium *	0.250 (0.199-0.301)[a]	0.250 (0.191-0.286)[a]	0.280 (0.233-0.338)[b]
Iron *	0.290 (0.229-0.369)[a]	0.272 (0.214-0.356)[a]	0.327 (0.259-0.416)[b]
Zinc *	0.277 (0.209-0.358)[a]	0.268 (0.196-0.339)[a]	0.317 (0.251-0.397)[b]
Manganese **	0.294 (0.238-0.371)[a]	0.309 (0.243-0.367)[a]	0.338 (0.271-0.405)[b]
Folate	0.336 (0.251-0.443)[a]	0.318 (0.247-0.416)[ab]	0.360 (0.285-0.466)[b]
Vitamin C	0.487 (0.333-0.706)	0.476 (0.329-0.690)	0.501 (0.375-0.742)
Vitamin A	0.540 (0.387-0.807)	0.527 (0.364-0.800)	0.560 (0.386-0.840)
Vitamin D	0.782 (0.583-1.044)	0.754 (0.512-1.013)	0.768 (0.581-0.971)
Vitamin E *	0.345 (0.259-0.434)[a]	0.359 (0.276-0.444)[ab]	0.395 (0.314-0.488)[b]
Vitamin K	0.569 (0.460-0.694)	0.576 (0.480-0.689)	0.593 (0.473-0.719)
Beta carotene *	0.746 (0.578-0.960)[a]	0.800 (0.626-1.006)[ab]	0.843 (0.652-1.080)[b]
Saturated fatty acids **	0.379 (0.313-0.455)[a]	0.398 (0.337-0.507)[b]	0.432 (0.359-0.519)[b]
Sodium (Salt) **	0.309 (0.261-0.364)[a]	0.315 (0.261-0.362)[a]	0.334 (0.288-0.395)[b]
Sugar **	0.396 (0.303-0.484)[a]	0.376 (0.304-0.465)[a]	0.440 (0.358-0.554)[b]
Phosphorus **	0.211 (0.174-0.262)[a]	0.223 (0.183-0.260)[a]	0.251 (0.206-0.313)[b]

When significant differences were observed between groups by the Kruskal-Wallis test, a post hoc Bonferroni-corrected Dunn's test was performed. Values with different superscript letters indicate significant differences ($p < 0.05$), whereas those sharing the same letter are not significantly different. Association was further analyzed using a generalized linear model adjusted for age, BMI and physical activity. *, $p < 0.05$; **, $p < 0.001$.

Table S5. Sensitivity analysis: additional adjustment for mean snack energy contribution (%E) and the day-to-day variability of snack energy intake (CV)

Nutrients	Estimate	95% CI (Lower)	95% CI (Upper)	*p* value
Carbohydrate **	0.150	0.086	0.213	0.0000
Fat **	0.120	0.064	0.177	0.0000
Protein **	0.127	0.067	0.187	0.0000
n-3 polyunsaturated fatty acids (PUFA)**	0.086	0.038	0.135	0.0005
Dietary fiber *	0.067	0.019	0.116	0.0063
Calcium *	0.077	0.029	0.125	0.0017
Magnesium **	0.118	0.061	0.174	0.0001
Potassium **	0.105	0.050	0.161	0.0002
Iron **	0.120	0.057	0.182	0.0002
Zinc **	0.120	0.049	0.190	0.0009
Manganese **	0.114	0.057	0.172	0.0001
Folate *	0.076	0.006	0.146	0.0330
Vitamin C	0.045	−0.050	0.140	0.3535
Vitamin A	0.024	−0.081	0.129	0.6568
Vitamin D	0.000	−0.071	0.072	0.9921
Vitamin E *	0.118	0.032	0.204	0.0075
Vitamin K	0.032	−0.019	0.084	0.2215
Beta carotene *	0.085	0.022	0.148	0.0088
Saturated fatty acids **	0.097	0.047	0.146	0.0001
Sodium (Salt) **	0.075	0.032	0.118	0.0006
Sugar **	0.122	0.069	0.176	0.0000
Phosphorus **	0.151	0.102	0.199	0.0000

As a sensitivity analysis, the association of mealtime regularity with the CV in each nutrient intake was analyzed using a generalized linear model adjusted for age, BMI, physical activity, snack energy contribution (%E), and day-to-day variability of snack energy intake. *, $p < 0.05$; **, $p < 0.001$.

Table S6. The ratio to reference daily value by Chronotype, Mealtime Regularity, Breakfast Timing, and Dinner Timing.

(a) Chronotype

		Median (Q1-Q3)		
**Nutrients**		**Morning**	**Intermediate**	**Evening**
Recommended nutrients	Protein	1.421 (1.243-1.742)	1.423 (1.269-1.725)	1.452 (1.267-1.666)
	n-3 polyunsaturated fatty acids (PUFA)	1.109 (0.859-1.362)	1.141 (0.864-1.421)	1.106 (0.852-1.339)
	Dietary fiber *	1.487 (1.257-1.796)	1.421 (1.216-1.752)	1.393 (1.111-1.662)
	Potassium **	1.238 (1.069-1.478)	1.212 (1.045-1.477)	1.150 (1.011-1.329)
	Calcium	1.252 (0.962-1.599)	1.223 (0.950-1.511)	1.209 (0.923-1.582)
	Magnesium	1.212 (1.039-1.560)	1.249 (1.016-1.492)	1.185 (0.992-1.465)
	Iron	1.517 (1.112-2.288)	1.419 (1.117-1.963)	1.367 (1.002-1.916)
	Zinc	1.200 (1.059-1.618)	1.205 (1.066-1.532)	1.239 (1.096-1.695)
	Manganese	1.045 (0.879-1.277)	1.025 (0.891-1.243)	0.985 (0.848-1.176)
	Folate	2.200 (1.625-2.870)	2.096 (1.658-2.762)	2.085 (1.528-2.816)
	Vitamin C	2.134 (1.401-3.264)	1.943 (1.325-2.901)	2.033 (1.307-3.197)
	Vitamin A	1.508 (1.030-2.234)	1.392 (0.976-2.156)	1.472 (0.989-2.340)
	Vitamin D	1.359 (0.912-2.197)	1.191 (0.813-1.924)	1.236 (0.896-1.937)
	Vitamin E	2.229 (1.665-3.290)	2.147 (1.611-3.043)	2.137 (1.669-3.229)
	Vitamin K *	2.006 (1.451-2.784)	2.060 (1.424-2.767)	1.850 (1.316-2.379)
Nutrients to be limited	Saturated fatty acids *	1.165 (0.929-1.355)	1.171 (1.000-1.347)	1.260 (1.066-1.465)
	Sodium (Salt)	1.538 (1.353-1.771)	1.599 (1.377-1.781)	1.582 (1.361-1.802)

(b) Mealtime regularity

		Median (Q1-Q3)	
**Nutrients**		**Regular**	**Irregular**
Recommended nutrients	Protein	1.425 (1.261-1.716)	1.457 (1.272-1.691)
	n-3 polyunsaturated fatty acids (PUFA)	1.132 (0.865-1.390)	1.125 (0.838-1.334)
	Dietary fiber	1.429 (1.192-1.752)	1.393 (1.183-1.735)
	Potassium	1.209 (1.040-1.435)	1.149 (1.050-1.409)
	Calcium	1.245 (0.958-1.573)	1.191 (0.923-1.515)
	Magnesium	1.236 (1.006-1.534)	1.179 (1.004-1.430)
	Iron	1.432 (1.101-2.045)	1.368 (1.007-1.998)
	Zinc	1.219 (1.075-1.621)	1.196 (1.056-1.602)
	Manganese	1.018 (0.863-1.228)	1.019 (0.870-1.246)
	Folate	2.163 (1.645-2.839)	2.026 (1.568-2.816)
	Vitamin C	2.045 (1.334-3.191)	1.994 (1.317-3.200)
	Vitamin A	1.442 (0.972-2.268)	1.420 (1.019-2.179)
	Vitamin D	1.275 (0.864-2.011)	1.236 (0.861-1.972)
	Vitamin E	2.145 (1.648-3.142)	2.257 (1.678-3.328)
	Vitamin K	1.989 (1.438-2.714)	1.885 (1.325-2.556)
Nutrients to be limited	Saturated fatty acids	1.189 (1.000-1.370)	1.213 (1.008-1.408)
	Sodium (Salt) *	1.604 (1.387-1.793)	1.503 (1.323-1.761)

Continued

Continued

(c) Breakfast timing

Nutrients		Median (Q1-Q3) Pre7am	7-8am	8-9am	Post9am
Recommended nutrients	Protein	1.382 (1.238-1.613)	1.444 (1.259-1.708)	1.406 (1.255-1.641)	1.501 (1.318-1.746)
	n-3 polyunsaturated fatty acids (PUFA)	1.130 (0.855-1.389)	1.094 (0.845-1.378)	1.152 (0.871-1.385)	1.141 (0.884-1.331)
	Dietary fiber	1.480 (1.231-1.798)	1.384 (1.183-1.696)	1.455 (1.176-1.797)	1.433 (1.222-1.708)
	Potassium	1.246 (1.076-1.470)	1.188 (1.031-1.408)	1.158 (1.019-1.431)	1.208 (1.070-1.409)
	Calcium	1.216 (0.947-1.522)	1.239 (0.945-1.568)	1.236 (0.925-1.541)	1.174 (0.972-1.574)
	Magnesium	1.225 (1.021-1.502)	1.208 (0.993-1.536)	1.250 (1.018-1.477)	1.196 (1.005-1.428)
	Iron	1.384 (1.151-1.971)	1.437 (1.094-2.025)	1.419 (1.075-2.134)	1.406 (1.036-1.929)
	Zinc	1.157 (1.061-1.385)	1.255 (1.080-1.718)	1.223 (1.060-1.637)	1.202 (1.074-1.529)
	Manganese	1.054 (0.878-1.270)	1.016 (0.862-1.267)	1.019 (0.861-1.259)	1.011 (0.867-1.170)
	Folate	2.125 (1.617-2.842)	2.121 (1.599-2.806)	2.068 (1.531-2.874)	2.187 (1.709-2.880)
	Vitamin C	2.083 (1.325-3.252)	2.001 (1.325-3.248)	2.052 (1.387-3.111)	1.988 (1.352-3.157)
	Vitamin A	1.416 (0.996-2.149)	1.391 (0.964-2.162)	1.418 (0.969-2.199)	1.641 (1.062-2.429)
	Vitamin D	1.186 (0.791-2.005)	1.275 (0.862-2.029)	1.247 (0.873-1.899)	1.405 (0.866-1.976)
	Vitamin E	2.192 (1.614-3.024)	2.183 (1.646-3.328)	2.064 (1.651-3.107)	2.182 (1.717-3.142)
	Vitamin K	2.146 (1.500-2.873)	1.925 (1.365-2.610)	1.893 (1.332-2.569)	1.948 (1.454-2.572)
Nutrients to be limited	Saturated fatty acids	1.183 (0.961-1.368)	1.199 (1.032-1.376)	1.190 (0.977-1.371)	1.227 (1.015-1.449)
	Sodium (Salt)	1.595 (1.357-1.753)	1.585 (1.394-1.784)	1.514 (1.321-1.786)	1.601 (1.348-1.814)

(d) Dinner timing

Nutrients		Median (Q1-Q3) Pre6pm	6-7pm	7-8pm	8-9pm	Post9pm
Recommended nutrients	Protein	1.382 (1.226-1.753)	1.465 (1.282-1.731)	1.417 (1.255-1.681)	1.457 (1.266-1.716)	1.429 (1.262-1.691)
	n-3 polyunsaturated fatty acids (PUFA)	1.012 (0.811-1.292)	1.129 (0.837-1.394)	1.126 (0.877-1.396)	1.142 (0.829-1.355)	1.188 (0.905-1.381)
	Dietary fiber *	1.448 (1.188-1.800)	1.453 (1.256-1.838)	1.417 (1.181-1.690)	1.335 (1.084-1.670)	1.324 (1.052-1.602)
	Potassium *	1.180 (1.033-1.374)	1.253 (1.072-1.460)	1.177 (1.039-1.402)	1.184 (0.995-1.400)	1.146 (0.995-1.330)
	Calcium **	1.261 (1.039-1.764)	1.299 (0.992-1.645)	1.191 (0.940-1.544)	1.172 (0.881-1.327)	1.220 (0.882-1.368)
	Magnesium	1.267 (1.018-1.673)	1.245 (1.050-1.581)	1.205 (1.005-1.480)	1.173 (0.974-1.410)	1.128 (0.990-1.423)
	Iron **	1.712 (1.121-2.312)	1.439 (1.147-2.153)	1.421 (1.078-2.001)	1.234 (0.965-1.648)	1.262 (0.965-1.730)
	Zinc	1.307 (1.078-1.775)	1.221 (1.073-1.556)	1.204 (1.073-1.607)	1.167 (0.985-1.428)	1.238 (1.098-1.686)
	Manganese	1.116 (0.879-1.359)	1.024 (0.856-1.238)	1.007 (0.881-1.181)	1.042 (0.856-1.279)	1.010 (0.812-1.222)
	Folate	2.129 (1.612-3.068)	2.206 (1.570-2.864)	2.142 (1.677-2.762)	1.829 (1.549-2.580)	1.856 (1.483-2.693)
	Vitamin C	2.023 (1.330-3.215)	2.123 (1.344-3.148)	2.008 (1.380-3.149)	1.898 (1.209-4.063)	1.772 (1.133-2.871)
	Vitamin A	1.614 (1.018-2.310)	1.452 (1.007-2.374)	1.440 (0.980-2.175)	1.284 (0.949-2.100)	1.381 (1.016-2.269)
	Vitamin D *	1.190 (0.831-1.960)	1.336 (0.925-2.397)	1.268 (0.855-1.898)	1.057 (0.709-1.910)	1.314 (0.897-1.862)
	Vitamin E	2.269 (1.757-3.559)	2.261 (1.698-3.224)	2.137 (1.649-3.229)	1.935 (1.506-3.106)	2.024 (1.498-2.645)
	Vitamin K	1.762 (1.301-2.448)	1.933 (1.450-2.698)	1.990 (1.402-2.733)	1.801 (1.353-2.349)	1.918 (1.362-2.622)
Nutrients to be limited	Saturated fatty acids	1.209 (0.997-1.402)	1.190 (1.000-1.357)	1.181 (0.996-1.379)	1.200 (1.041-1.431)	1.275 (1.091-1.481)
	Sodium (Salt)	1.465 (1.269-1.762)	1.581 (1.393-1.782)	1.600 (1.388-1.800)	1.477 (1.293-1.748)	1.621 (1.404-1.788)

When significant differences were observed between groups by the Kruskal-Wallis test (or Mann-Whitney U test), association was further analyzed using a generalized linear model adjusted for age, BMI and physical activity. *, $p < 0.05$; **, $p < 0.001$

(A)

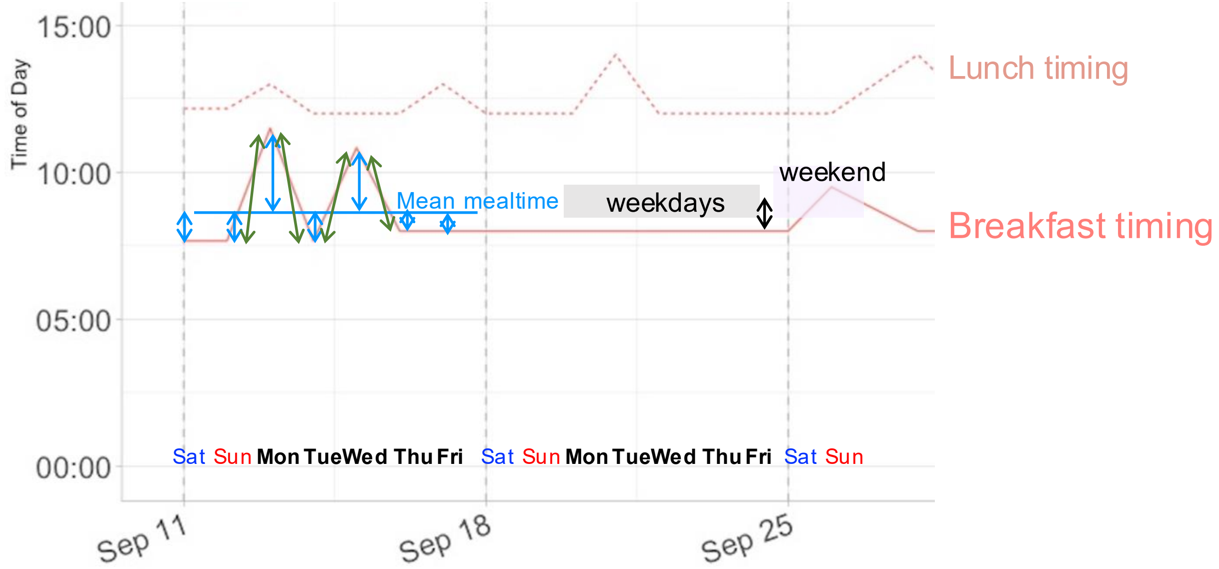

$$CPD = \frac{1}{N}\sum_{i=1}^{N}\sqrt{(T_i - T_{i-1})^2 + (T_i - Mean\ time)^2}$$

N, i, T denotes the total number of days, a given day, and mealtime, respectively.

As described by McHill *et al.* (Sci Rep, 2020), CPD is calculated based on both the difference in mealtime from the previous day and the deviation from the individual's mean mealtime, rather than solely comparing the average values between weekdays and weekends.

(B)

Representative patterns of the regular mealtime group

Representative patterns of the irregular mealtime group

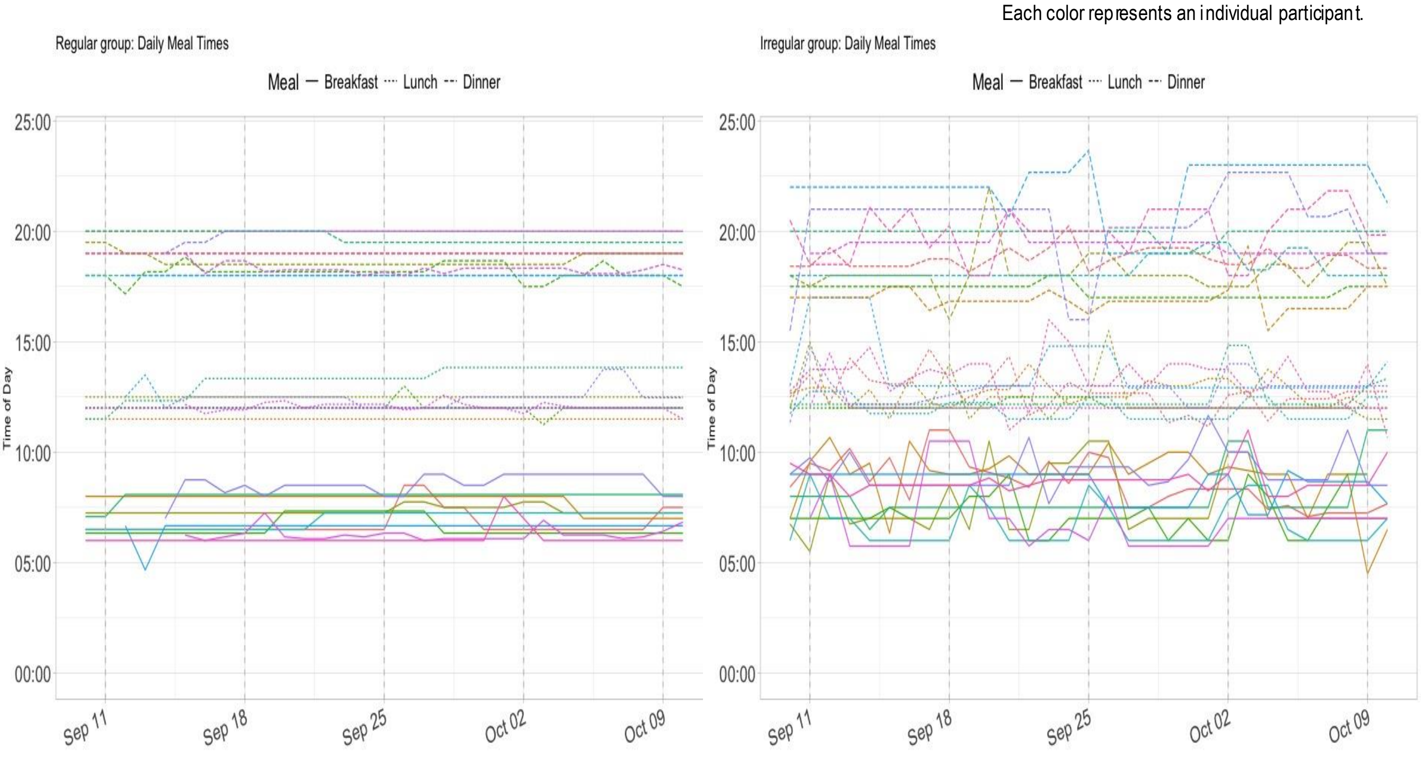

Figure S1. Conceptual illustration of the Composite Phase Deviation (CPD) (A) and representative 1-month mealtime patterns (B)

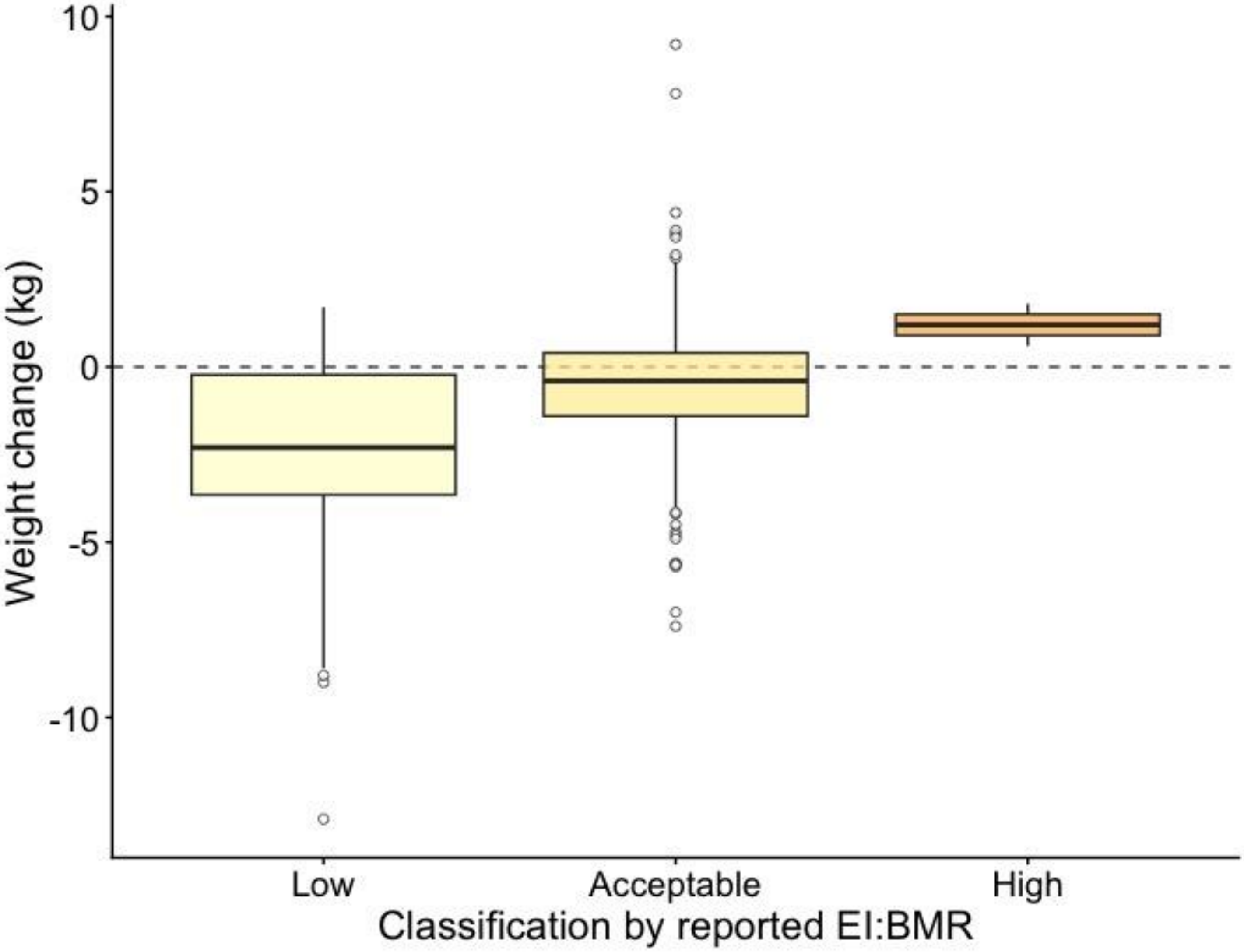

Figure S2. Weight Change by EI:BMR Ratio group.

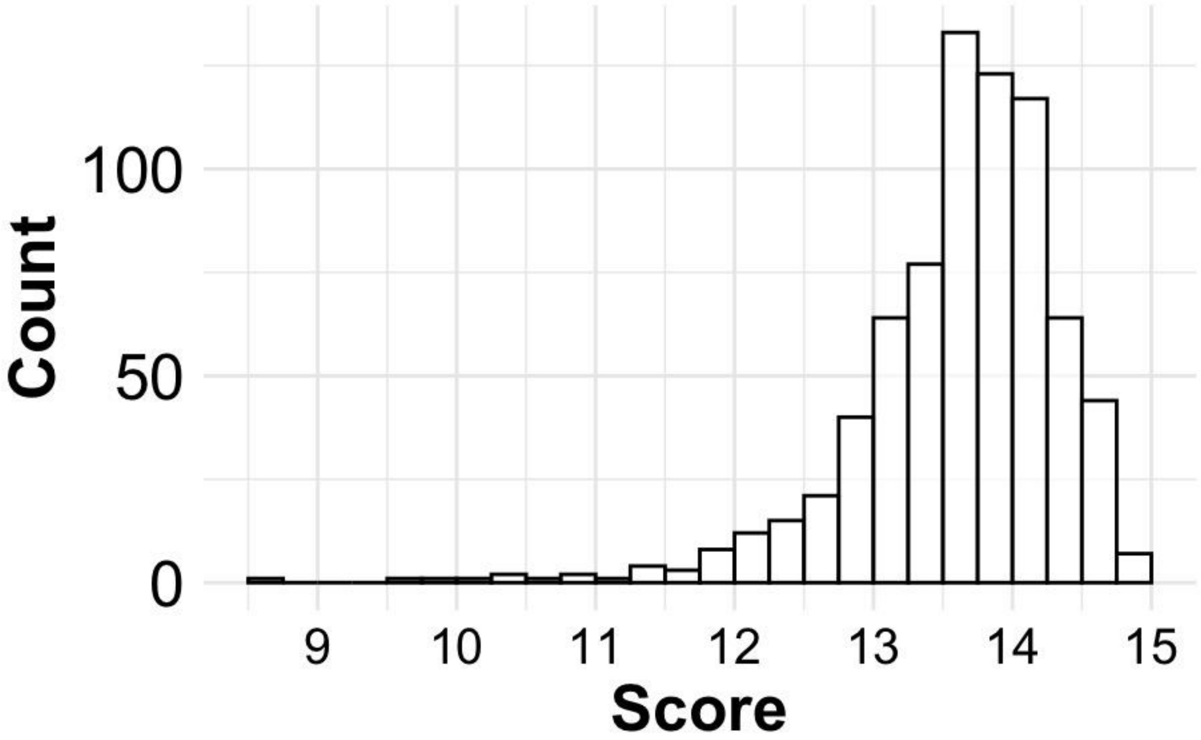

Figure S3. Nutritional score distribution.
